# Independent association of serum IgA levels with moderate-to-severe obstructive sleep apnea in children with adenotonsillar hypertrophy

**DOI:** 10.3389/fped.2026.1791876

**Published:** 2026-05-08

**Authors:** Fei Xia, Zhongfang Xia, Tao Huang, Dong Li

**Affiliations:** Department of Otolaryngology-Head and Neck Surgery, Wuhan Children's Hospital, Tongji Medical College, Huazhong University of Science & Technology, Wuhan, Hubei, China

**Keywords:** adenotonsillar hypertrophy, biomarkers, children, immunoglobulin A, obstructive sleep apnea

## Abstract

**Background:**

Adenotonsillar hypertrophy (ATH) is the leading cause of obstructive sleep apnea (OSA) in children. While the role of allergic inflammation (IgE-mediated) has been widely studied, the contribution of mucosal immunity—specifically Immunoglobulin A (IgA)—to disease severity remains unclear. This study aimed to investigate the association between serum immune markers and OSA severity and to construct a multivariate diagnostic model for moderate-to-severe disease.

**Methods:**

A total of 110 children diagnosed with ATH were enrolled in this cross-sectional study. Based on polysomnography (PSG) results and the *2020 Chinese Guidelines for Diagnosis and Treatment of Childhood OSA*, participants were divided into a Mild/No OSA group (*n* = 65) and a Moderate-to-Severe OSA group (*n* = 45). Serum immunoglobulins (IgA, IgE, IgG, IgM) and complement components (C3c, C4) were analyzed. Logistic regression and Receiver Operating Characteristic (ROC) curve analyses were performed.

**Results:**

The Moderate-to-Severe group exhibited significantly lower lowest oxygen saturation (LSpO2) [0.85 (0.81, 0.89) vs. 0.89 (0.86, 0.91), *P* < 0.001], larger adenoids [A/N ratio: 0.77 (0.71, 0.80) vs. 0.65 (0.62, 0.75), *P* < 0.001], and a higher proportion of Grade III tonsils (28.9% vs. 12.3%, *P* < 0.001). Notably, serum IgA levels were significantly elevated in the Moderate-to-Severe group (2.05 ± 0.55 vs. 1.79 ± 0.75 g/L, *P* = 0.032), while IgE showed no significant difference. Multivariate logistic regression identified LSpO2 (OR = 0.853, *P* = 0.005) as a protective factor, while Tonsillar Hypertrophy (ORs 27.09–45.54) and IgA (OR = 2.520, 95% CI: 1.033–6.146, *P* = 0.042) were independent risk factors. A combined model incorporating these variables achieved an Area Under the Curve (AUC) of 0.852.

**Conclusion:**

Elevated serum IgA is an independent associated factor of moderate-to-severe OSA in children with ATH, suggesting a potential mechanism involving chronic mucosal inflammation distinct from atopy. A combined diagnostic model involving IgA, anatomical metrics, and oximetry provides high diagnostic value.

## 1 Introduction

Obstructive sleep apnea (OSA) in the pediatric population is a prevalent disorder characterized by prolonged partial upper airway obstruction and/or intermittent complete obstruction during sleep, disrupting normal ventilation and sleep architecture (1). Recent epidemiological data indicate that the prevalence of childhood OSA in China is approximately 4.8%, comparable to global estimates of 1.2%–5.7% (2, 3). Unlike adult OSA, which is strongly associated with obesity, the primary etiology in children is adenotonsillar hypertrophy (ATH) (3, 4). If left untreated, the chronic intermittent hypoxia and sleep fragmentation caused by OSA can lead to severe long-term sequelae, including neurocognitive deficits, behavioral abnormalities, cardiovascular dysfunction, and growth retardation (5, 6).

Currently, the “gold standard” for diagnosing and stratifying OSA severity is overnight polysomnography (PSG) (7). However, PSG is resource-intensive, expensive, and not readily available in all medical centers, leading to significant diagnostic delays. Furthermore, the clinical decision for adenotonsillectomy is often complicated by the fact that tonsil size alone correlates poorly with the physiological severity of obstruction (8). Some children with massive lymphoid hyperplasia exhibit only mild symptoms, while others with moderate hypertrophy suffer from severe desaturation. This discrepancy suggests that underlying pathophysiological mechanisms—potentially immunological in nature—play a critical role in modulating airway patency and collapsibility.

The tonsils and adenoids are secondary lymphoid organs of Waldeyer's ring, serving as the first line of defense in the upper respiratory tract. Existing literature has extensively explored the “Unified Airway” theory, focusing on the role of Immunoglobulin E (IgE) and allergic sensitization in ATH (9–11). However, a significant proportion of children with severe OSA are non-atopic, and recent evidence suggests that chronic inflammation driven by recurrent viral or bacterial infections may be a more potent driver of refractory hypertrophy (12, 13). Immunoglobulin A (IgA), particularly its secretory form, is crucial for mucosal immunity. Yet, compared to IgE, the potential of serum IgA as a biomarker for OSA severity has been understudied, although elevated serum IgA levels have been observed in children with OSA, indicating an associated chronic inflammatory state (14).

We hypothesize that systemic elevation of IgA reflects a chronic inflammatory state of the upper airway mucosa that contributes to the severity of OSA, independent of allergic status. To test this, we conducted a cross-sectional study involving 110 children with ATH. We strictly applied the *2020 Chinese Guidelines for Diagnosis and Treatment of Childhood OSA* (15) to categorize severity and employed multivariate statistical models to determine whether serum IgA, alongside anatomical and hypoxic markers, acts as an independent risk factor for moderate-to-severe disease.

## 2 Methods

### 2.1 Study design and ethical considerations

This was a retrospective cross-sectional study conducted at the Department of Otolaryngology-Head and Neck Surgery of our hospital from March 2021 to December 2023. The study protocol was approved by the Institutional Review Board of our hospital. Written informed consent was obtained from the parents or legal guardians of all participants. The study is reported in accordance with the Strengthening the Reporting of Observational Studies in Epidemiology (STROBE) guidelines (16).

### 2.2 Participants

We enrolled children aged 3–15 years who presented with symptoms of sleep-disordered breathing (SDB), such as snoring, mouth breathing, or witnessed apnea, and were diagnosed with adenotonsillar hypertrophy via nasopharyngoscopy or lateral neck radiography. A total of 110 children were included in the final analysis.

Inclusion criteria: (1) Age between 3 and 15 years; (2) Confirmed diagnosis of ATH; (3) Completion of standard overnight PSG monitoring; (4) Availability of complete serological immune profile data.

Exclusion criteria: (1) Craniofacial anomalies (e.g., Pierre Robin sequence, Down syndrome); (2) Neuromuscular disorders; (3) Acute infection within the last 4 weeks; (4) History of autoimmune diseases or immunodeficiency; (5) Use of systemic corticosteroids or immunomodulators within the past 3 months; (6) Previous upper airway surgery.

### 2.3 Polysomnography and grouping

Overnight PSG was performed using a standard computerized system (e.g., Alice 6, Philips Respironics) in a quiet, sleep-conducive laboratory. Measured parameters included electroencephalogram (EEG), electrooculogram (EOG), electromyogram (EMG), electrocardiogram (ECG), oronasal airflow, thoracoabdominal respiratory effort, and pulse oximetry (SpO2). Sleep stages and respiratory events were scored manually by certified technicians according to the American Academy of Sleep Medicine (AASM) Manual for the Scoring of Sleep and Associated Events (Pediatric Rules) (17).

The primary outcome metrics were the Obstructive Apnea-Hypopnea Index (OAHI) and the lowest oxygen saturation (LSpO2). According to the *Chinese Guidelines for Diagnosis and Treatment of Childhood Obstructive Sleep Apnea (2020)* (12), an OAHI > 1 event/h is considered diagnostic for OSA. Severity was classified as follows: Mild (1 < OAHI ≤ 5 events/h), Moderate (5 < OAHI ≤ 10 events/h), and Severe (OAHI > 10 events/h). In this study, to identify factors associated with clinically significant disease requiring intervention, participants were divided into two groups: the Mild/No OSA Group (OAHI ≤ 5 events/h, *n* = 65) and the Moderate-to-Severe OSA Group (OAHI > 5 events/h, *n* = 45).

### 2.4 Clinical and laboratory measurements

Anatomical Assessment: Tonsil size was graded using the Brodsky scale (0–4+) (18). For statistical analysis, tonsil size was categorized into three grades: Grade I (tonsils occupy <25% of the oropharyngeal width), Grade II (25%–50%), and Grade III (50%–75% or >75%, merging Brodsky 3+ and 4+ due to small sample size in extreme grades). Adenoid size was evaluated using the Adenoid-Nasopharyngeal (A/N) ratio derived from lateral cephalometric radiographs or endoscopy, recorded as a continuous variable (0.00–1.00).

Serological Assays: Fasting venous blood samples were collected in the morning following PSG. Serum levels of immunoglobulins (IgA, IgE, IgG, IgM) and complement components (C3c, C4) were quantified using Enzyme-Linked Immunosorbent Assay (ELISA) or nephelometry, strictly following the manufacturer's instructions. Unlike previous studies that dichotomized these values, we analyzed them as continuous variables to preserve statistical power and biological gradient.

### 2.5 Statistical analysis

Data were analyzed using SPSS 26.0 (IBM Corp., Armonk, NY, USA). Normality of continuous variables was assessed using the Shapiro–Wilk test. Normally distributed data were expressed as mean ± standard deviation (SD) and compared using the Student's *t*-test. Non-normally distributed data were presented as median (interquartile range, IQR) and compared using the Mann–Whitney *U*-test. Categorical variables were expressed as frequencies (percentages) and compared using the Chi-square-test.

Spearman's rank correlation analysis was used to explore the relationships between clinical parameters and immune markers. Multicollinearity was assessed using Variance Inflation Factor (VIF) and Tolerance. Variables with significant differences in univariate analysis (*P* < 0.1) and biologically plausible relevance were entered into a multivariate Binary Logistic Regression model to identify independent associated factors of moderate-to-severe OSA. The results were presented as Odds Ratios (OR) with 95% Confidence Intervals (CI).

The diagnostic performance of independent risk factors and the combined model was evaluated using Receiver Operating Characteristic (ROC) curve analysis. The Area Under the Curve (AUC) was calculated, and the optimal cutoff values were determined using the Youden index. A two-tailed *P*-value < 0.05 was considered statistically significant.

## 3 Results

### 3.1 Baseline characteristics

The study included 110 children. As shown in Table 1, 65 children (59.1%) were in the Mild/No OSA group, and 45 (40.9%) were in the Moderate-to-Severe OSA group. There were no statistically significant differences in age, gender distribution, or BMI between the two groups (*P* > 0.05).

**Table 1:** Comparison of baseline characteristics between groups.

Variable	Mild/No OSA (*n* = 65)	Moderate-to-severe OSA (*n* = 45)	Test stat (*t*/*Z*/*χ*^2^)	*P*-value
Age (years)	8.77 ± 2.21	8.28 ± 2.11	1.173	0.243
Gender (Male/Female)	46/19	35/10	0.673	0.412
BMI (kg/m^2^)	15.70 (14.50, 21.15)	16.40 (14.05, 22.15)	0.265	0.791
LSpO2 (%)	0.89 (0.86, 0.91)	0.85 (0.81, 0.89)	3.487	<0.001
Tonsil Size (*n*)			7.398	<0.001
Grade I	11 (16.9%)	2 (4.4%)		
Grade II	46 (70.8%)	30 (66.7%)		
Grade III	8 (12.3%)	13 (28.9%)		
Adenoid Size (A/N)	0.65 (0.62, 0.75)	0.77 (0.71, 0.80)	4.858	<0.001
IgE (IU/mL)	86.50 (34.90, 211.00)	61.40 (26.20, 266.00)	0.310	0.757
IgA (g/L)	1.79 ± 0.75	2.05 ± 0.55	2.147	0.032
IgM (g/L)	1.23 ± 0.45	1.08 ± 0.36	1.367	0.171
IgG (g/L)	12.05 ± 2.59	11.75 ± 2.39	0.561	0.576
C3c (g/L)	1.11 ± 0.21	1.16 ± 0.21	1.220	0.226
C4 (g/L)	0.22 ± 0.08	0.24 ± 0.08	1.033	0.304

Values are mean ± SD, median (IQR), or *n* (%). LSpO2, Lowest Oxygen Saturation; Ig, Immunoglobulin; C, Complement.

**Figure 1:**
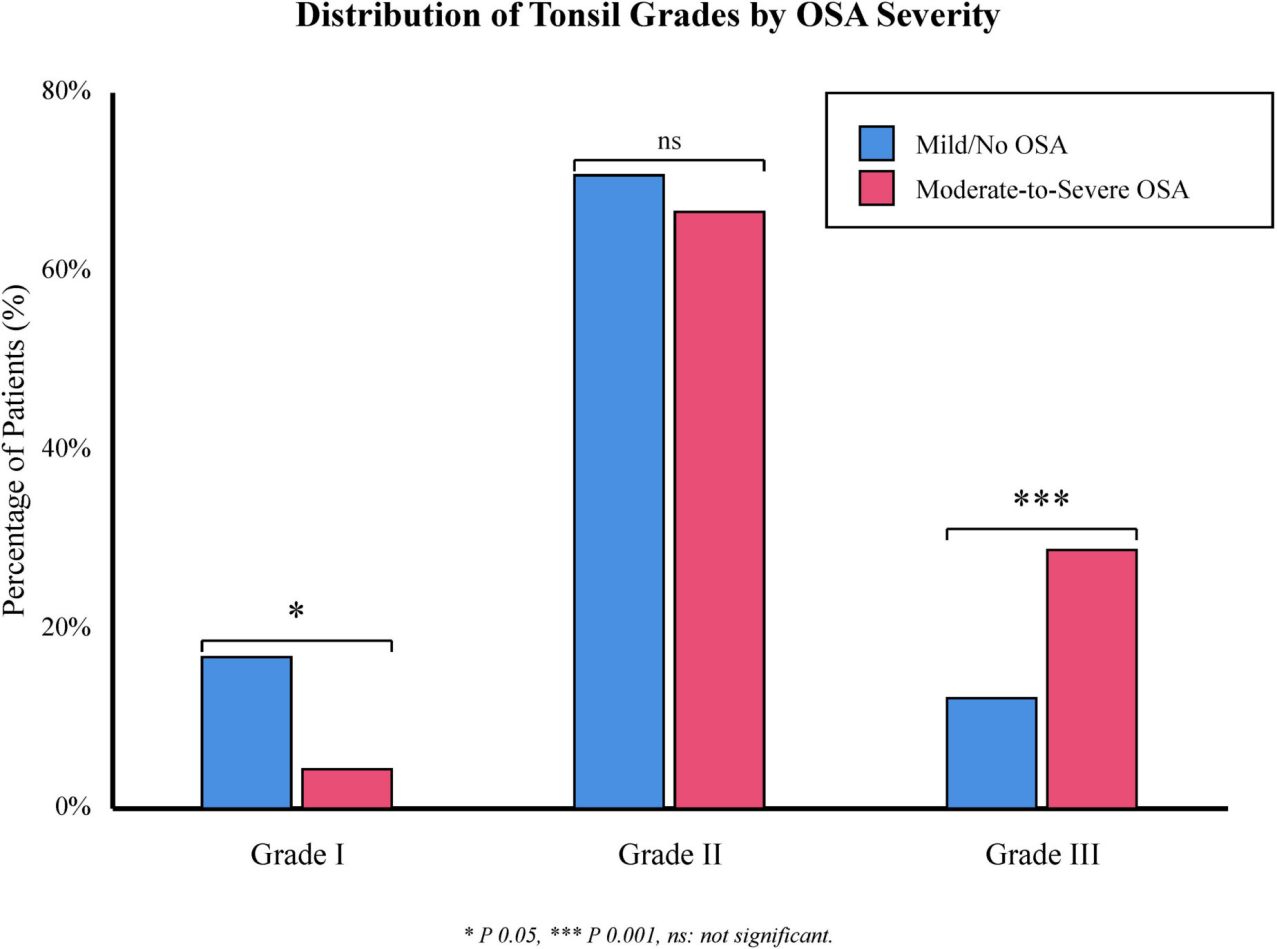
Distribution of tonsillar hypertrophy grades by OSA severity. The prevalence of tonsillar hypertrophy grades differs significantly between groups. The moderate-to-severe OSA group (dark bars) exhibits a markedly higher proportion of Grade III tonsils (28.9%) compared to the Mild/No OSA group (light bars, 12.3%), while Grade I tonsils are predominantly found in the Mild/No OSA group (16.9% vs. 4.4%). *P < 0.05.

Regarding anatomical and physiological parameters, the Moderate-to-Severe group exhibited significantly lower LSpO2 compared to the Mild/No group [0.85 (0.81, 0.89) vs. 0.89 (0.86, 0.91), *Z* = 3.487, *P* < 0.001]. Both adenoid size (A/N ratio) and tonsil grade were significantly higher in the Moderate-to-Severe group (*P* < 0.001). Specifically, the proportion of Grade III tonsils was 28.9% in the severe group vs. only 12.3% in the mild group, as illustrated in Figure 1.

In terms of immune profiles, serum IgA levels were significantly elevated in the Moderate-to-Severe group (2.05 ± 0.55 g/L) compared to the Mild/No group (1.79 ± 0.75 g/L) (*t* = 2.147, *P* = 0.032). Other markers, including IgE, IgM, IgG, C3c, and C4, did not show statistically significant differences between groups.

### 3.2 Correlation analysis

**Figure 2:**
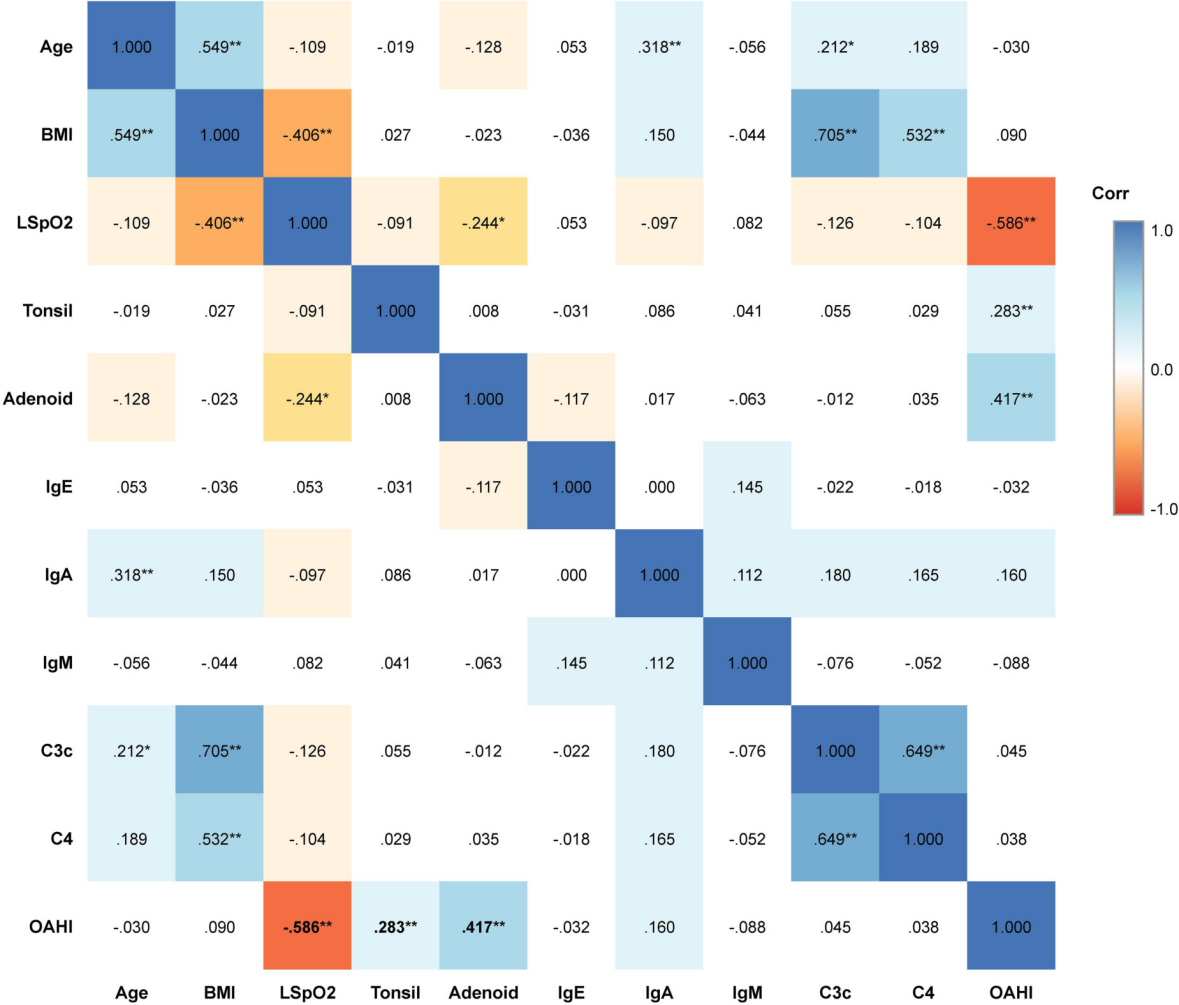
Spearman correlation heatmap of Key clinical and immunological variables. The heatmap displays the pairwise Spearman correlation coefficients **(r)** for the study population (*n* = 110). Colors represent the strength and direction of the correlation: blue indicates a positive correlation, while red indicates a negative correlation. The intensity of the color corresponds to the magnitude of the coefficient, as shown in the legend bar on the right. Numerical values represent the correlation coefficient **(r)**. Statistical significance is denoted by asterisks: *P < 0.05, **P < 0.01. OAHI, Obstructive Apnea-Hypopnea Index; LSpO2, Lowest Oxygen Saturation; C3c, Complement component 3c; C4, Complement component 4; Ig, Immunoglobulin.

Spearman's rank correlation analysis (Table 2 and Figure 2) was performed to explore relationships between clinical and immunological variables. The Apnea-Hypopnea Index (AHI), the primary marker for disease severity, showed a significant negative correlation with LSpO2 (*r* = −0.586, *P* < 0.01) and significant positive correlations with Adenoid Size (*r* = 0.417, *P* < 0.01) and Tonsil Size (*r* = 0.283, *P* < 0.01).

**Table 2:** Spearman Correlation Coefficients among Key Variables.

Variables	Age	BMI	LSpO2	Tonsil	Adenoid	IgE	IgA	IgM	C3c	C4	OAHI
Age	1	.549^b^	−.109	−.019	−.128	.053	.318^b^	−.056	.212^a^	.189	−.030
BMI	.549^b^	1	−.406^b^	.027	−.023	−.036	.150	−.044	.705^b^	.532^b^	.090
LSpO2	−.109	−.406^b^	1	−.091	−.244^a^	.053	−.097	.082	−.126	−.104	−.586^b^
Tonsil	−.019	.027	−.091	1	.008	−.031	.086	.041	.055	.029	.283^b^
Adenoid	−.128	−.023	−.244^a^	.008	1	−.117	.017	−.063	−.012	.035	.417^b^
IgE	.053	−.036	.053	−.031	−.117	1	.000	.145	−.022	−.018	−.032
IgA	.318^b^	.150	−.097	.086	.017	.000	1	.112	.180	.165	.160
IgM	−.056	−.044	.082	.041	−.063	.145	.112	1	−.076	−.052	−.088
C3c	.212^a^	.705^b^	−.126	.055	−.012	−.022	.180	−.076	1	.649^b^	.045
C4	.189	.532^b^	−.104	.029	.035	−.018	.165	−.052	.649^b^	1	.038
OAHI	−.030	.090	−.586^b^	.283^b^	.417^b^	−.032	.160	−.088	.045	.038	1

a Correlation is significant at the 0.05 level (2-tailed).

b Correlation is significant at the 0.01 level (2-tailed). *n* = 110.

Regarding immune markers, IgA showed a moderate positive correlation with Age (*r* = 0.318, *P* < 0.01), reflecting the maturation of the immune system. Significant internal correlations were also observed, such as BMI with C3c (*r* = 0.705) and C4 (*r* = 0.532), and strong correlations between complement components (C3c and C4, *r* = 0.649), validating the internal consistency of the biological data. In contrast, IgE and IgM did not show significant correlations with OSA severity indices (AHI or LSpO2).

### 3.3 Multicollinearity diagnosis

Prior to multivariate regression, a collinearity diagnostic was performed (Table 3). The Tolerance values for all independent variables (Sex, BMI, Age, LSpO2, Tonsil, Adenoid, IgE, IgA, IgM, C3c, C4) ranged from 0.330 to 0.914, all greater than 0.1. The Variance Inflation Factors (VIF) ranged from 1.094 to 3.029, all well below the threshold of 5. These results indicate that there was no significant multicollinearity among the independent variables, ensuring the stability of the regression model.

**Table 3:** Collinearity diagnostics for multivariate analysis (excerpt).

Model	Tolerance	VIF
Gender	0.663	1.508
BMI	0.330	3.029
Age	0.599	1.669
LSpO2	0.693	1.443
Tonsil Size	0.862	1.160
Adenoid Size	0.871	1.148
IgE	0.914	1.094
IgA	0.840	1.191

VIF, Variance Inflation Factor. A VIF < 5 indicates no significant multicollinearity.

### 3.4 Independent risk factors for moderate-to-severe OSA

To identify the independent determinants of OSA severity, we constructed a multivariate binary logistic regression model. The dependent variable was the severity grouping (Mild/No OSA vs. Moderate-to-Severe OSA). Covariates entered into the model included Age, Gender, LSpO2, Tonsil Size, Adenoid Size, IgM, and IgA. The results, detailed in Table 4, revealed a distinct risk profile for severe disease.

**Table 4:** Multivariate logistic regression analysis for moderate-to-severe OSA.

Variable	B	Sig. (P)	OR (Exp B)	95% CI for OR
Age	−0.299	0.053	0.742	0.548–1.004
Gender (Male)	0.038	0.955	1.039	0.271–3.989
LSpO2	−0.159	**0**.**005**	0.853	0.764–0.953
Tonsil Size		**0**.**015**		
Grade II (vs. I)	3.299	0.015	27.088	1.903–385.583
Grade III (vs. I)	3.819	0.013	45.537	2.265–915.376
Adenoid Size	5.950	0.057	383.825	0.829–1.77E6
IgM	−1.027	0.194	0.358	0.076–1.685
IgA	0.924	**0**.**042**	2.520	1.033–6.146
Constant	7.701	0.173	2,210.3	

OR, Odds Ratio; CI, Confidence Interval. Significant *P*-values (<0.05) are highlighted in bold.

Three independent associated factors reached statistical significance:

1. **LSpO2 (Protective Factor):** The lowest oxygen saturation was significantly negatively associated with disease severity (OR = 0.853, 95% CI: 0.764–0.953, *P* = 0.005). This indicates that for every 1% decrease in LSpO2, the odds of having moderate-to-severe OSA increase by approximately 17% (1/0.853).
2. **Tonsil Size (Major Risk Factor):** Anatomical obstruction by the tonsils was the most potent associated factor. Using Grade I as the reference, children with Grade II tonsils had an Odds Ratio (OR) of 27.09 (*P* = 0.015), and those with Grade III tonsils had an OR of 45.54 (*P* = 0.013).
3. **Serum IgA (Novel Biomarker):** Crucially, serum IgA emerged as an independent risk factor even after adjusting for anatomical and hypoxic variables. For every 1 g/L increase in serum IgA, the risk of moderate-to-severe OSA increased by 2.52-fold (OR = 2.520, 95% CI: 1.033–6.146, *P* = 0.042).

Variables such as Age, Gender, and IgM did not show statistical significance (*P* > 0.05). Notably, while Adenoid Size was significant in univariate analysis, it showed only borderline significance in the multivariate model (*P* = 0.057), suggesting its effect might be partially captured by other variables or is less critical than tonsillar size in this specific cohort.

### 3.5 Diagnostic performance (ROC analysis)

**Figure 3:**
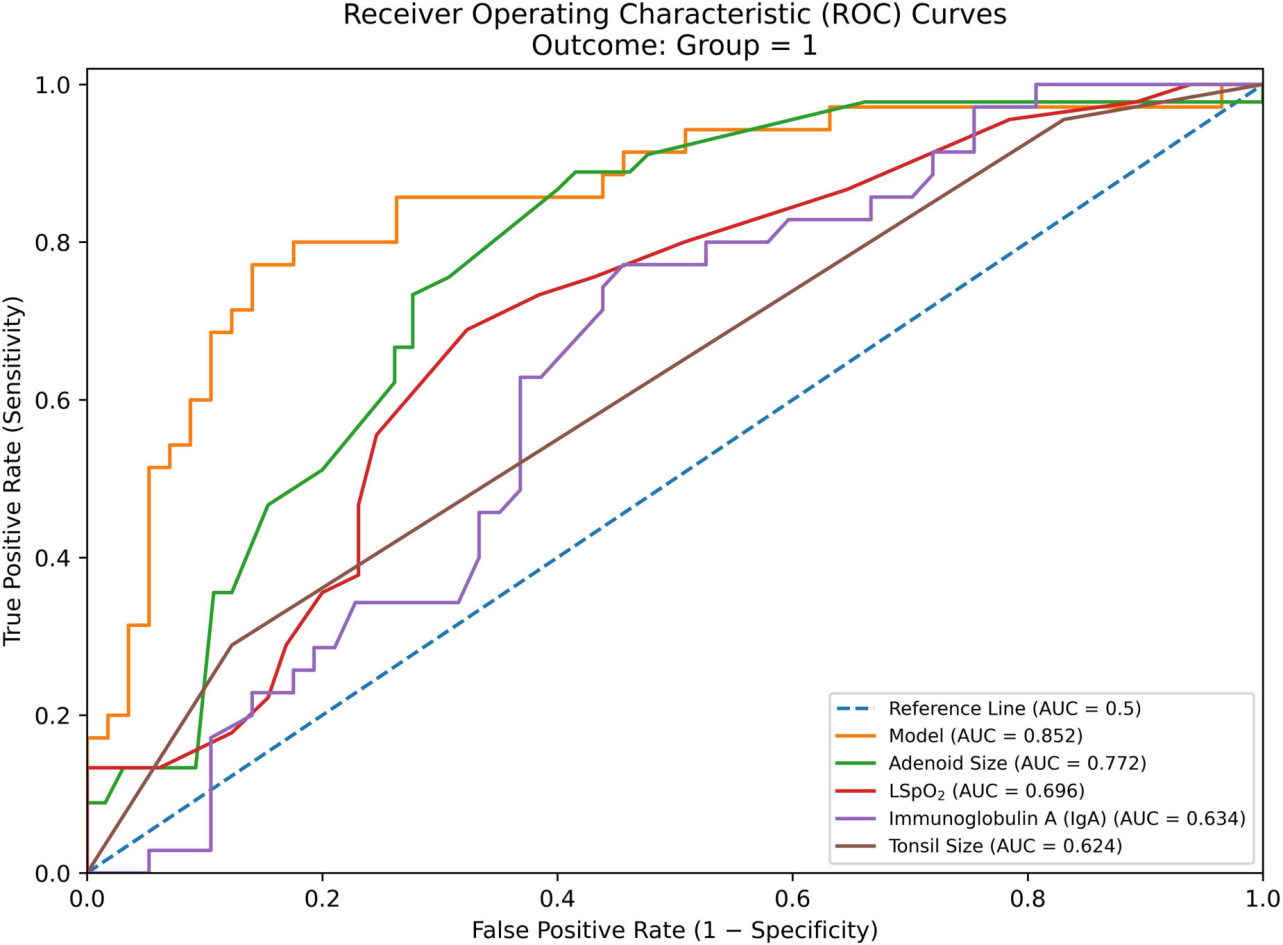
Receiver operating characteristic (ROC) curves for identifying moderate-to-severe OSA. The ROC curves illustrate the diagnostic performance of individual biomarkers and the combined diagnostic model. The **Model** (red solid line), which integrates serum IgA, LSpO2, Tonsil Size, and Adenoid Size, demonstrates the highest diagnostic accuracy with an Area Under the Curve (AUC) of 0.852 (*P* < 0.001). Among single indicators, **Adenoid Size** (blue dashed line, AUC = 0.772) and **LSpO2** (green dashed line, AUC = 0.696) show moderate efficacy. **Serum IgA** (yellow dashed line, AUC = 0.634) provides significant independent diagnostic value. In contrast, **Age** and **IgM** (gray dotted lines) hover near the diagonal reference line, indicating no significant discriminative ability.

To evaluate the translational potential of these findings, Receiver Operating Characteristic (ROC) curve analysis was performed (Table 5 and Figure 3). Among single parameters, Adenoid Size exhibited the highest diagnostic accuracy (AUC = 0.772, *P* < 0.001), followed by LSpO2 (AUC = 0.696, *P* < 0.001) and IgA (AUC = 0.634, *P* = 0.021). Consistent with regression findings, Age and IgM provided no significant diagnostic value.

**Table 5:** Diagnostic performance of individual and combined indicators (ROC analysis).

Variable	AUC	S.E.	Sensitivity (%)	Specificity (%)	Youden Index	*P*-value
Age	0.570	0.056	64.44	52.31	0.168	0.211
Tonsil Size	0.624	0.043	28.89	87.69	0.166	0.004
Adenoid Size	0.772	0.045	88.89	58.46	0.474	<0.001
IgA	0.634	0.058	77.14	54.39	0.315	0.021
IgM	0.585	0.061	97.14	19.30	0.164	0.161
LSpO2	0.696	0.051	68.89	67.69	0.366	<0.001
Model	0.852	0.043	77.14	85.96	0.631	<0.001

SE, standard error. Model = Combined diagnostic model utilizing LSpO2, Tonsil Size, Adenoid Size, and IgA.

A combined probabilistic model (“Model”) was generated based on the logistic regression equation. This composite model achieved a superior AUC of 0.852 (95% CI: 0.768–0.936, *P* < 0.001). At the optimal Youden index of 0.631, the model demonstrated a sensitivity of 77.14% and a specificity of 85.96%, significantly enhancing the capability to identify children with moderate-to-severe OSA compared to any single biomarker.

## 4 Discussion

This study provides a comprehensive investigation into the immunological and anatomical determinants of OSA severity in children with adenotonsillar hypertrophy (ATH). By strictly adhering to the *2020 Chinese Guidelines* (15), we classified patients into clinically relevant groups and demonstrated that moderate-to-severe OSA is driven by a complex interplay of tonsillar obstruction, hypoxic burden, and, most notably, elevated serum IgA levels. Our findings challenge the traditional focus on IgE-mediated allergy and highlight a potential “chronic infectious/inflammatory” phenotype in severe pediatric OSA. The composite diagnostic model developed here (AUC = 0.852) offers a robust, non-invasive tool for risk stratification.

### 4.1 The “silent” inflammation: IgA as a marker of chronic biofilm infection

The most pivotal finding of this study is that elevated serum IgA acts as an independent risk factor for moderate-to-severe OSA (OR = 2.52), while IgE does not. This distinction is crucial. Historically, ATH has been strongly linked to allergic rhinitis and atopy, mediated by IgE (19). However, our data show no significant difference in total IgE levels between mild and severe groups (*P* = 0.757). This suggests that while allergy may contribute to initial lymphoid proliferation or nasal resistance, it may not be the primary driver of the *severe, collapsible* airway obstruction seen in advanced OSA.

In contrast, IgA is the predominant immunoglobulin of the mucosal immune system. We speculate that the elevated serum IgA in severe OSA patients reflects a spillover from a highly active local immune response in the tonsils and adenoids. Recent microbiological studies continue to highlight the relevance of dense bacterial biofilms in pediatric tonsillar disease (20). These biofilms act as chronic reservoirs for pathogens, providing persistent antigenic stimulation that drives follicular hyperplasia and germinal center expansion, as seen in OSA tonsils compared to those from recurrent tonsillitis (20). Unlike acute infection (marked by IgM, which was non-significant in our study), this chronic biofilm-associated inflammation leads to a sustained upregulation of IgA production. Furthermore, chronic inflammation can induce structural changes that alter upper airway tissue compliance, making it more prone to collapse during sleep, a relationship underscored by studies linking tonsil size to increased airway negative pressure (21). Thus, our finding of elevated IgA supports the evolving hypothesis that severe OSA in non-atopic children is a manifestation of chronic, infection-driven mucosal immune activation rather than a purely allergic process (22) Specifically, while IgM typically characterizes acute phase responses, the significant elevation of serum IgA likely reflects a sustained, adaptive mucosal defense against persistent intrafollicular antigenic stimulation prevalent in OSA-related hypertrophy.

### 4.2 Hypoxia and the vicious cycle of inflammation

Our multivariate analysis identified LSpO₂ as a significant independent associated factor (OR = 0.853), reinforcing the critical role of hypoxia. Intermittent hypoxia is not merely a downstream consequence of apnea but an active pathogenic operator. At the cellular level, intermittent hypoxia mimics ischemia-reperfusion injury, activating the NF-*κ*B pathway and promoting the release of systemic inflammatory cytokines, such as TNF-α and IL-6 (23). This systemic inflammation can, in turn, further stimulate lymphoproliferation in Waldeyer's ring, creating a vicious cycle where obstruction causes hypoxia, hypoxia drives inflammation, inflammation promotes further lymphoid hypertrophy, and worse obstruction ensues (24, 25). The significant negative correlation we observed between OAHI and LSpO₂ (*r* = −0.586) aligns with previous reports confirming that desaturation depth is a reliable proxy for event frequency and physiological stress (26).

### 4.3 Anatomical dominance: tonsils vs. Adenoids

While adenoid hypertrophy is a known risk factor, our multivariate model highlighted tonsillar hypertrophy as the dominant anatomical risk factor for severe disease (OR > 27 for Grade II/III). This aligns with the “Starling resistor” model of the upper airway, where the collapsible pharyngeal segment, particularly the oropharynx bounded by the tonsils, is a key site of obstruction (27). Adenoids primarily obstruct the nasopharynx, causing obligate mouth breathing (28). While mouth breathing alters airway dynamics and predisposes to collapse, as noted in reviews of its role in sleep-disordered breathing, the tonsils directly encroach upon the narrowest part of the airway. Interestingly, adenoid size was a strong screener (AUC = 0.772) but lost significance to tonsils in the regression, suggesting that in severe cases, the “choke point” moves downstream to the tonsillar level. This has practical implications for surgical planning, supporting tonsillectomy (with or without adenoidectomy) as the definitive treatment for severe pediatric OSA, consistent with recent outcomes research that affirms its role as first-line therapy (7).

### 4.4 Clinical utility of the combined model

The waiting time for polysomnography (PSG) in many pediatric centers can exceed several months, highlighting an urgent need for a reliable triage tool (29). Our combined model, integrating Serum IgA, LSpO2, Tonsil Size, and Adenoid Size, achieved an AUC of 0.852 with a high specificity of ∼86%. The inclusion of IgA significantly enhanced the model's discriminative power, as evidenced by the increase in AUC from 0.772 (for Adenoid Size alone) to 0.852 (for the combined model). This performance is superior to using the Brodsky scale or SpO2 alone.

In clinical practice, this model implies that a child presenting with Grade III tonsils, a history of significant desaturation (low LSpO₂), and elevated serum IgA is highly likely to have moderate-to-severe OSA. Such patients could be prioritized for early surgical intervention (“fast-track pathway”) or expedited PSG. Conversely, a child with large tonsils but normal IgA and normal oximetry might be managed more conservatively or with anti-inflammatory medication (e.g., intranasal corticosteroids) first, as part of the medical treatment options for pediatric OSA (30).

### 4.5 Limitations and future directions

Several limitations warrant mention. First, the cross-sectional design precludes establishing causality; we cannot confirm if elevated IgA causes hypertrophy or results from it. Therefore, IgA should be viewed as an associated factor rather than a strict diagnostic tool. Longitudinal studies tracking IgA levels post-adenotonsillectomy would be valuable. Second, we measured total serum IgA. While clinically accessible, assessing secretory IgA in saliva or tonsillar tissue homogenates could offer more direct insights into the local immune environment implicated in tonsil hyperplasia (20). Third, although we excluded acute infections, subclinical viral carriage was not tested. Fourth, our findings indicated a positive correlation between IgA and age, suggesting age may be a residual confounding factor despite its inclusion in the regression model, a common limitation in pediatric cohorts of this size. Finally, while combining Mild and No OSA groups introduces some clinical heterogeneity, this binary classification maximizes the statistical power to distinguish patients who are clear candidates for surgical intervention (moderate-to-severe) from those manageable via observation or medical therapy, consistent with clinical triaging priorities.

## 5 Conclusion

This study identifies serum IgA as a novel, independent biomarker associated with the severity of Obstructive Sleep Apnea in children with adenotonsillar hypertrophy. The association of elevated IgA with severe disease, independent of tonsil size and hypoxia, suggests a distinct pathophysiological pathway likely involving chronic biofilm-mediated mucosal inflammation. We propose a composite risk stratification model combining IgA, oximetry, and anatomical grading, which demonstrates high diagnostic accuracy. These findings support the inclusion of immunological profiling in the routine assessment of pediatric OSA to guide personalized therapeutic strategies.

## Data Availability

The original contributions presented in the study are included in the article/Supplementary Material, further inquiries can be directed to the corresponding author/s.
